# Psychological Correlates of (Non)Use of Formal Resources by Family Caregivers of People With Dementia

**DOI:** 10.1093/geroni/igab046.1743

**Published:** 2021-12-17

**Authors:** Laura Gallego-Alberto, Isabel Cabrera, María Márquez-González, María del Sequeros Pedroso Chaparro, Laura Mérida-Herrera, Cristina Huertas, Andrés Losada-Baltar

**Affiliations:** 1 Universidad Autónoma de Madrid, Madrid, Madrid, Spain; 2 Universidad Rey Juan Carlos, Madrid, Madrid, Spain

## Abstract

Caring for a relative with dementia is a stressful task characterized by a high number of demands extended in time. Therefore, caregivers frequently report the need for assistance to cope with the situation. However, formal resources use is low among that population. The objective of this study was to explore the correlates of (non)use of formal resources (day care center and home care) by family caregivers of people with dementia. Participants were 225 dementia family caregivers that were individually assessed in a) use of formal resources, b) sociodemographic variables, c) stressors (frequency and reaction to behavioral problems), and d) psychological variables (depression, anxiety, and dysfunctional thoughts about caregiving). A logistic regression was done comparing those who used formal resources with those who did not use them. Caregivers who did not use formal resources were younger (OR = .95; 95% CI [.92 - .98]), devoted more daily hours to caring (OR = 1.07; 95% CI [1.02 - 1.11]), reported higher levels of dysfunctional thoughts about caregiving (OR = 1.07; 95% CI [1.04 – 1.10]) and higher anxiety levels (OR = 1.07; 95% CI [1.00- 1.13]), and their care-recipient had a higher functional autonomy (OR = 1.04; 95% CI [1.02 – 1.05]). Higher levels of anxiety and dysfunctional thoughts in caregivers may be act as barriers to seek for formal support. Targeting these variables may help to increase the use of formal resources by family caregivers of people with dementia.

